# Organic waste and beechwood cellulose blend saccharification and validation of hydrolysates by fermentation

**DOI:** 10.1007/s00253-024-13349-2

**Published:** 2024-11-14

**Authors:** Stanislav Rudnyckyj, Sergey Kucheryavskiy, Tanmay Chaturvedi, Mette Hedegaard Thomsen

**Affiliations:** 1 Department of Energy, Aalborg University, Niels Bohrs Vej 8, 6700 Esbjerg, Denmark; 2 Department of Chemistry and Bioscience, Aalborg University, Niels Bohrs Vej 8, 6700 Esbjerg, Denmark

**Keywords:** OFMSW, Biomass blends, Enzymatic hydrolysis, Ethanol fermentation, Biomass production, Natural buffer, Nitrogen substitution

## Abstract

**Abstract:**

This study demonstrates the sustainable advancement of fermentation media by blending the organic fraction of municipal solid waste (OFMSW) with organosolv beechwood cellulose. Investigations examined the effects of enzyme dosages and OFMSW integration into organosolv beechwood cellulose on sugar yield. The findings indicate that OFMSW inclusion and Cellic® CTec3 dosage significantly influence hydrolysis across two different batches of beechwood cellulose. Experimental data showed that OFMSW inclusion levels of 35% and 45% (w/w) produced sugar levels comparable to pure beechwood cellulose, achieving 58% to 68% (w/w) saccharification with sugar concentrations of 44 to 46 g/L. This highlights OFMSW's potential as a buffer substitute during the enzymatic conversion of organosolv cellulose. The resulting sugar-rich hydrolysates, derived from OFMSW-cellulose blends and pure cellulose, were evaluated for ethanol and cell biomass production using *Saccharomyces cerevisiae* and *Mucor indicus*, yielding 30 g of ethanol/L hydrolysate. Furthermore, OFMSW inclusion in beechwood cellulose proved to be an excellent alternative to synthetic nitrogen agents for *S. cerevisiae* cell production, reaching 12.2 g of biomass/L and surpassing the biomass concentration from cultivation on cellulose hydrolysate with nitrogen supplementation by threefold. However, *M. indicus* did not grow in the OFMSW-cellulose blend, suggesting that the inhibitory compounds of OFMSW may be a bottleneck in the proposed process. The present study demonstrates the benefits of incorporating OFMSW into cellulose material, as it enhances both cost-effectiveness and sustainability. This is attributed to the natural buffering properties and nitrogen content of OFMSW, which reduces the need for synthetic agents in fermentation-based lignocellulose biorefineries.

**Key points:**

• *OFMSW inclusion significantly influences beechwood cellulose saccharification.*

• *OFMSW could be an excellent alternative for synthetic agents in biorefinery.*

• *S. cerevisiae achieved higher biomass growth on OFMSW/cellulose mix compared to YPD.*

**Graphical abstract:**

**Figure:**
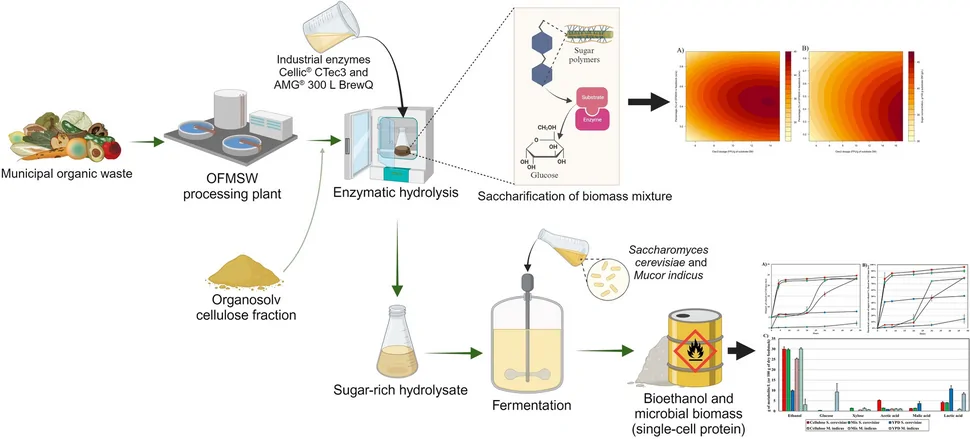

**Supplementary Information:**

The online version contains supplementary material available at 10.1007/s00253-024-13349-2.

## Introduction

Abundant, low-cost, and renewable organic materials like the OFMSW and lignocellulosic residues, such as beechwood sawdust, serve as promising second-generation feedstocks for biofuel and bulk chemicals production. Despite advancements in recycling practices for organic material management in the EU, landfilling and incineration still account for 23% and 26% of OFMSW utilization, respectively, resulting in considerable environmental harm (Eurostat 2022). Repurposing OFMSW into a combined biorefinery with lignocellulose could redirect current harmful practices toward more sustainable applications.

Both types of biomass are rich in fermentable sources, particularly sugars. OFMSW typically contains around 19% w/w cellulose, 9% w/w hemicellulose, 17% w/w of starch, and 11% w/w of free sugars on a dry basis (Campuzano and González-Martínez 2016), and pretreated beechwood sawdust consists of approximately 88% w/w of cellulose and 7% w/w of hemicellulose on a dry basis (Buzala et al. 2019). However, cellulose and hemicellulose are components of the lignocellulosic fraction, which possesses a robust structure, making it highly resistant to biological conversions. Therefore, a pretreatment step is required to extract monomeric sugars from biomass. Pretreatment processes are necessary to break down the complex lignocellulose matrix, releasing fermentable sugars, mainly glucose and xylose, and enabling further bioconversion (Thomsen et al. 2008; Ashraf et al. 2017; Ebrahimian et al. 2023). These pretreatments usually are based on mechanical, chemical, physiochemical, and biological methods. Generally, mechanical pretreatments are used as the first step for size reduction, resulting in an increase in surface area, making it more accessible for chemical and biological interactions (Kumari and Singh 2018).

In the case of OFMSW, it is wise to consider heat treatment for enhancing the solubility of organic compounds, like proteins and sugars, and the dissolution of the integrity of the biomass cell wall (Petersen et al. 2009; Rudnyckyj et al. 2023). Additionally, OFMSW may contain a relatively high starch content, exceeding 20% on a dry basis (Hansen et al. 2007). Therefore, as part of heat pretreatment, starch gelatinization could be necessary as it would swell starch and consequently make it accessible for microbial and/or enzymatic conversion (Parchami et al. 2021). Moreover, thermal treatment can serve as a sterilization step (Chen et al. 2012).

Generally, OFMSW has a relatively high concentration of inhibitors, which originate from the high content of phenolic compounds in fruit, vegetable, tea, and coffee waste residuals (Ebrahimian et al. 2023), metals (Dornau et al. 2020), and microbial metabolites such as alcohols, organic acids, and aldehydes (Shao et al. 2014; Jojoa-Unigarro and González-Martínez 2022). The detoxification step could be considered, as it can be crucial for the process if enzymes and sensitive microorganisms are used (Jönsson et al. 2013). However, the preference lies in using robust fermentative microorganisms that can tolerate and/or metabolize the inhibitors without the need for additional detoxification, ensuring a more efficient and economically viable biochemical production process.

Regarding the processing of beechwood sawdust, a hardwood lignocellulose material, common pretreatment methods prior to enzymatic cellulose hydrolysis include acidic treatment, hydrothermal processing, mild alkali treatment, ionic liquid pretreatment, and chemical pulping processes (Jönsson and Martín 2016). As an alternative to traditional chemical pulping, organosolv pretreatment has been developed (Vaidya et al. 2022). This approach involves the use of organic solvents to extract lignin and hemicellulose from the biomass (Thoresen et al. 2020), leading to subsequent simultaneous pretreatment and detoxification of the cellulose-rich material (Farmanbordar et al. 2018; Ebrahimian and Karimi 2020).

Enzymatic hydrolysis (EH) is the extensively used and widely researched pretreatment method for processing residual biomass. However, due to the complexity, recalcitrance, and heterogeneous character of biowastes, particularly OFMSW, the enzymatic hydrolysis process requires the use of industrial hydrolytic enzyme mixtures based on cellulases, hemicellulases, amylases, and other enzymes to effectively break down fibers into fermentable sugars (Paritosh et al. 2018). In general, the application of industrial enzymes has demonstrated promising outcomes in the saccharification of both OFMSW and beechwood Table 1.

**Table 1:** Current development in EH of OFMSW and delignified beechwood pulp

Type of biomass	Used enzymes	Sugar yield (%) and concentration (g/L)	Reference
OFMSW	Viscozyme L® (Novozymes A/S) cocktail containing arabanase, cellulase, β-glucanase, hemicellulase, and xylanase with loading of 100 Fungal Beta Glucanase Units per g of substrate	51%, 50 g/L	Molina-Peñate et al. 2022
OFMSW	Stargen™ 002 (DuPont) with the loading of 0.01% (v/v)	25%, 49 g of glucose/L	Haske-Cornelius et al. 2020
OFMSW	*Trichoderma reesei* cellulase (Sigma-Aldrich) with the loading of 90 mg/g of substrate	53%, 32 g/L	Li et al. 2012
OFMSW	cellulase complex, amylase, hemicellulase, pectate lyase, lipase, and protease (Novozymes A/S)	60%, 140 g of glucose/L	Nwobi et al. 2015
OFMSW	Cellic® CTec2 (Novozymes A/S) with loading of 20 FPU/g OFMSW dry matter and amylases (Novozymes A/S) with loading of 0.1 g per g of starch in OFMSW	82%, 41 g glucose/L	Ebrahimian et al. 2020
OFMSW	Pentopan 500 BG, Celluclast BG, and Glucoamylase NS 22035 (Novozymes A/S) with loading of 90, 150, and 108 mg/g of substrate	56%, 24 g/L	Izaguirre et al. 2019
OFMSW	Cellic® CTec2 (Novozymes A/S), Cellic® Htec2 (Novozymes A/S) with loading of 20 FPU/g OFMSW dry matter and α-amylase Liquezyme (Novozymes A/S) and glucoamylase Dextrozyme GA (Novozymes A/S) with loading of 2 g per kg of starch in OFMSW	70 g/L	Mahmoodi et al. 2018
OFMSW	Cellulases and amylases (Novozymes A/S)	70 g/L	López-Gómez et al. 2019
Organosolv beechwood pulp	Cellic® CTec2 (Novozymes A/S) with loading of 8.4 mg/g of substrate	40%, 75 g glucose/L	Kalogiannis et al. 2018
Organosolv beechwood pulp	Celluclast® (Novozymes A/S) with the loading of 32.5 FPU/g of substrate	60%, 12 g/L	Regestein et al. 2018
Organosolv beechwood pulp	Celluclast® (Novozymes A/S) with the loading of 32.5 FPU/g of substrate	55%	Wang et al. 2017
Organosolv beechwood pulp	Cellic® CTec2 (Novozymes A/S), Cellic® Htec2 (Novozymes A/S) with loading of 5 FPU/g of substrate	29%, 151 g glucose/L	Tippkötter et al. 2014
Acetone/water oxidation beechwood pulp	Cellic® CTec2 (Novozymes A/S) with loading of 9 mg/g of substrate	55%, 121 g glucose/L	Katsimpouras et al. 2017

However, the combined enzymatic conversion of OFMSW and organosolv pretreated beechwood sawdust mixtures has not been observed in previous studies and is yet to be investigated. Existing research primarily concentrates on the anaerobic digestion of OFMSW and lignocellulose blends, with a particular focus on their impact on biogas production (Tyagi et al. 2018). Using OFMSW with cellulosic biomass offers several advantages, particularly in regulating pH and contamination control. Cellulosic materials often require the use of buffering agents to maintain an optimal pH range for efficient hydrolysis with cellulolytic enzymes, which is typically between a pH of 4–6 (Payne et al. 2015). OFMSW can substitute traditional buffers because its pH falls within a similar range (Campuzano and González-Martínez 2016), and its organic acids and salts provide natural buffering properties (Cheah et al. 2019). Additionally, to promote optimal microbial growth during the fermentation of cellulosic hydrolysates, a nitrogen source is typically required, which can increase costs and reduce the overall sustainability of the technology. However, the inclusion of OFMSW can serve as an alternative nitrogen source, due to its relatively high protein and nitrogen-rich salt content (Rajpal et al. 2014; Campuzano and González-Martínez 2016). Lastly, despite both biomasses being categorized as second-generation feedstocks, OFMSW is more abundant, easier to process sustainably, and consequently more cost-effective compared to delignified cellulose, which requires more severe conditions and chemicals for processing. Incorporating OFMSW into cellulosic biomass has the potential to significantly enhance cost-effectiveness throughout the entire production process, making it an appealing alternative to conventional approaches.

Therefore, the primary objective of this research was to validate the anticipated benefits of combining OFMSW and beechwood cellulose as an efficient and eco-friendly approach for the enzymatic conversion of second-generation feedstocks into fermentable sugars, which can serve as a production platform for biofuels and bulk chemicals. To evaluate the suitability of sugar-rich hydrolysates as cultivation media, ethanol fermentation and biomass production were applied. Additionally, all fermentations were conducted under non-sterile conditions to verify contamination control from antimicrobial compounds in the OFMSW.

## Materials and methods

### Materials

OFMSW was collected in October 2022 at Holsted municipality (55° 30′ 41″ N, 8° 54′ 59″ E), Denmark, and provided by Ragn-Sells Denmark A/S. The OFMSW was stored at − 80 °C till further use. Luleå University of Technology provided extracted cellulose from beechwood sawdust. Conditions for organosolv treatment were 180 °C for 60 min with 60% (v/v) ethanol and 20 mM H_2_SO_4_ (liquid-to-solid-ratio of 10; v/w). The enzymatic mixture was based on Cellic® CTec3 (Novozymes A/S) as a source of cellulases and hemicellulases with specific enzymatic activity 214 FPU/mL and AMG® 300 L BrewQ (Novozymes A/S) as a source of glucoamylase with specific enzymatic activity 300 AGU/mL. In this study, Cellic® CTec3 and AMG® 300 L BrewQ are referred to as Ctec3 and AMG, respectively. Sugars, organic acids, and ethanol were analyzed through high-performance liquid chromatography (HPLC) (1260 Infinity II, Agilent Technologies). As fermentative organisms, *S. cerevisiae* (commercial dry yeast, Malteserkors tørgær, De Danske Spritfabrikker A/S, Denmark) and *M. indicus* DSM 2185 were used.

### Enzymatic hydrolysis

Prior to enzymatic hydrolysis, approximately 1 to 2 kg of OFMSW were defrosted at room temperature overnight and heat pretreated in an autoclave at 100 °C for 4 h (Rudnyckyj et al. 2023). The experimental procedure involved creating the solution by carefully measuring either beechwood cellulose or mixtures of OFMSW with beechwood cellulose in an analytical weight fashion within a 500-mL flask. Subsequently, Mili-Q water was added, followed by the addition of enzymes by direct pipetting as the final step. Based on the experimental design, the Ctec3 dosage ranged from 1 to 22.07 FPU/g of substrate dry matter (DM), and the AMG dosage ranged from 0.5 to 5 AGU/g of substrate DM. To achieve a final weight of 100 g, required quantities of OFMSW, beechwood cellulose, enzymatic solution, and Mili-Q water were added to 500 mL shaking flasks. In the case of EH of pure beechwood cellulose, 0.05 M acetate buffer at pH 5 was used instead of Mili-Q water. These flasks were then placed in an incubator and maintained at 50 °C and 150 RPM for 24 h. Incubated samples were removed, and each hydrolysate was poured into two 50-mL falcon tubes, one of which was stored at − 20 °C. The samples were centrifuged (SL 16 Centrifuge, Thermo Fisher Scientific) at 5000 RPM for 20 min. Five milliliters of supernatant was carefully piped out into new 50-mL falcon tubes. The supernatant was diluted by the addition of 20 mL of Mili-Q water. These diluted hydrolysates were then subjected to filtration using 0.22-μm syringe filters and transferred into HPLC vials for subsequent analysis of sugar content. For storage, the HPLC vials containing the hydrolysates were frozen at − 20 °C. Throughout the experiments, whether involving beechwood cellulose or mixtures of OFMSW with beechwood cellulose, a final concentration of 10% w/w DM was maintained, with necessary dilution using Mili-Q water. Equation 1 was used to calculate the addition of Mili-Q water:

1 $${\mathrm{m}}_{\mathrm{water}}=100\text{ g}-\left({\mathrm{m}}_{\mathrm{beechwood}}+{\mathrm{m}}_{\mathrm{OFMSW}}\right)-({\mathrm{V}}_{\mathrm{Ctec}3}{\uprho }_{\mathrm{Ctec}3}+ {\mathrm{V}}_{\mathrm{AMG}}{\uprho }_{\mathrm{AMG}})$$

where *m*_water_ is the mass of Mili-Q water that needs to be added to reach 100 g of total solution volume, *m*_beechwood_ is the mass of organosolv beechwood in the reaction mixture, *m*_OFMSW_ is the mass of OFMSW in the reaction mixture, *V*_Ctec3_ is the volume of added Ctec3 solution, *V*_AMG_ is the volume of added AMG solution, *ρ*_Ctec3_ is the density of Ctec3 solution, 1.2 g/mL, and *ρ*_AMG_ is the density of AMG solution, 1.16 g/mL.

### *M. indicus* inoculum preparation

The *M. indicus* DSM 2185 strain was streaked onto a YPD (yeast extract–peptone–dextrose) agar plate from a glycerol stock using an inoculation loop and then incubated at 30 °C for 6 days. Subsequently, mature *M. indicus* spores were rinsed with 40 mL of sterile Milli-Q water and gathered in a sterile 50-mL falcon tube. For inoculation, 10 mL of the spore solution per sample was directly pipetted into the fermentation medium.

### Ethanol fermentation

The sugar-rich hydrolysates, prepared with 15 FPU of Ctec3/g biomass DM and, in the case of mixtures of OFMSW and beechwood pulp cellulose, 0.5 AMG/g biomass DM, were supplemented with 0.20 g of *S. cerevisiae* or inoculum of *M. indicus*. Hydrolysates produced purely from beechwood cellulose were spiked with 1 mL of sterile nitrogen-rich solution (100 g/L of yeast extract and 20 g/L urea) as a source of nitrogen and growth factors. Nitrogen gas was used to flush prepared 500 mL flasks for 10–15 s to eliminate air, and yeast locks containing 2 mL glycerol were added. The incubation was performed under anaerobic conditions at 32 °C with 130 RPM for 96 h in a shaking incubator (LS-Z shaker with Kelvin^+^, Kuhner). The weight of each flask was measured before incubation and subsequently at 4, 8, 20, 30, and 48 h into the incubation. Following incubation, each fermentation broth was poured into two 50-mL falcon tubes, one of which was stored at − 20 °C. Samples were centrifuged (SL 16 Centrifuge, Thermo Fisher Scientific) at 5000 RPM for 20 min, and 5 mL of supernatant was carefully pipetted into new 50-mL falcon tubes. The supernatant was diluted with 20 mL of Mili-Q water, and the diluted hydrolysates were filtered with 0.22-μm syringe filters into HPLC vials and assessed for ethanol, free sugar, and organic acid content. The samples were frozen at − 20 °C for storage. Calculation of ethanol production from gravimetric determination, where MW is the molecular weight Eq. 2. Gravimetric measurements were verified by ethanol analysis via HPLC.

2 $$\text{EtOH }(\mathrm{g}) =\text{ C}{\mathrm{O}}_{2} (\mathrm{g}) \times [\text{MW }(\mathrm{EtOH})/[\text{MW }({\mathrm{CO}}_{2})]$$

### Microbial biomass production

The sugar-rich hydrolysates, prepared with 15 FPU of Ctec3/g biomass DM and, in the case of mixtures of OFMSW and beechwood pulp cellulose, 0.5 AMG/g biomass DM, underwent centrifugation (SL 16 Centrifuge, Thermo Fisher Scientific) at 4500 RPM for 20 min. Supernatants were collected, and 90 mL of slurry was transferred into each 250 mL shaking flask. For the hydrolysates originating solely from beechwood cellulose, 10 mL of a sterile nitrogen-rich solution (100 g/L of yeast extract and 20 g/L of urea) was added. Subsequently, fermentation media were supplemented with 0.20 g of *S. cerevisiae* or inoculum of *M. indicus*. The flasks were sealed using caps equipped with PTFE membranes. The incubation was performed under aerobic conditions at 32 °C with 130 RPM for 48 h in a shaking incubator (LS-Z shaker with Kelvin^+^, Kuhner). After incubation, each fermentation broth was centrifuged (SL 16 Centrifuge, Thermo Fisher Scientific) at 3000 RPM for 10 min. Supernatants were discarded, and pellets with cells were washed with 100 mL of Mili-Q water and centrifuged again. The washed cells were used to identify biomass production by drying at 60 °C overnight and weighing on analytical balance.

### Chemical analysis

DM, also often referred to as total solid, was determined in OFMSW samples based on the following analytical protocol by the National Renewable Energy Laboratory (NREL) (Sluiter et al. 2008a).

A CHNS elemental analyzer (Pekin Elmer 2400 series CHNS/O) was used to analyze the carbon, hydrogen, nitrogen, and sulfur content of the OFMSW samples. The samples were analyzed as a dried sample, where a 2–10-mg sample was weighed on small tin capsules and analyzed on the elemental analyzer.

For the total sugar determination, biomass material was dried in an oven at 60 °C overnight and after was powdered with a porcelain mortar. Strong acid hydrolysis was performed to identify the total sugar content using the analytical protocol developed by NREL (Sluiter et al. 2008b). The characterization of the sugars, ethanol, and organic acids in post-enzymatic hydrolysates and fermentation broths was based on the analytical protocol by NREL (Sluiter et al. 2006). Produced samples were filtered by 0.22-μm syringe filters into HPLC vials and underwent HPLC analysis (Bio-Rad Aminex HPX-87H Column, Bio-Rad Laboratories Inc.), using H_2_SO_4_ mobile phase (0.005 M) and RID (Refractive Index Detector) for sugar (glucose, cellobiose, xylose, and arabinose), ethanol, and acetic acid. The organic acids (malic, formic, and lactic acids) were analyzed, using H_3_PO_4_ mobile phase (0.2 v/v %) and DAD (Diode Array Detection). The mathematical conversion of HPLC results (g/L) into (g/100 g OFMSW DM) was performed as described elsewhere (Nwobi et al. 2015; Alassali et al. 2017). A hydration factor of 0.9 was used to estimate glucan content from glucose concentration, while a hydration factor of 0.88 was applied to estimate xylan and arabinan from pentoses.

For total starch content determination, OFMSW samples were prepared by drying 100 g in an oven at 105 °C overnight. Then, dried samples were powdered with a porcelain mortar and sieved with a 0.5-mm metal screen. The prepared samples were analyzed for starch content by Megazymes Total Starch HK Assay Kit (Megazyme Ltd.).

### Statistical methods

All trials were performed at least as triplicates, and the samples were prepared in random order. All results are given as mean values with standard deviation. The one-way analysis of variance (ANOVA) was carried out on all results with one independent variable, while N-way ANOVA was used for the analysis of screening experiments with multiple independent variables. The significance level was set to 0.05 for all performed statistical tests. Second-order multiple linear regression (MLR) was performed for the analysis of optimization experiments based on response surface methodology. ANOVA results were supplemented by Tukey’s honest significant difference (HSD) test when necessary. The MLR outcomes are presented graphically in the form of 2D contour plots, showing the dependence of response (dependent variable) on the factors under optimization (independent variables). R version 4.3.1 (R Core Team 2020) was used for all statistical analyses.

## Results

### The compositional analysis of OFMSW and organosolv cellulose from beechwood

The compositional analysis of the OFMSW and beechwood cellulose from batch 1 and batch 2 applied in this study are tabulated in Table 2. The DM for the OFMSW was 14.4% (w/w), and the pH was 4.02. The compositional analysis of beechwood cellulose from batch 1 and batch 2 reveals a significant variation in sugar content, deliberately selected to assess the reproducibility of the bioconversion outcomes. The difference in glucan content between the two beechwood cellulose batches is attributed to two factors: the natural variation in glucan content in beechwood and the standard variation in glucan recovery following organosolv pretreatment.

**Table 2:** The composition of biomasses is presented as g/100 g of biomass DM, except organic acids in OFMSW which are presented in g/L

*Component*	*OFMSW*	*Beechwood cellulose (batch 1)*	*Beechwood cellulose (batch 2)*
Carbon (%)	48.2 ± 1.4	42.0 ± 0.11	43.0 ± 0.06
Hydrogen (%)	6.77 ± 0.73	6.54 ± 1.45	5.89 ± 0.31
Nitrogen (%)	3.05 ± 0.11	0.9 ± 0.06	-
Sulfur (%)	0.43 ± 0.07	-	0.64 ± 0.03
*Total sugars*	42.8 ± 2.1	75.9 ± 0.5	97.5 ± 2.5
Glucan	38.3 ± 2.5	75.9 ± 0.5	97.5 ± 2.5
Xylan	4.42 ± 0.20	-	-
Arabinan	-	-	-
Starch	14.7 ± 0.9	-	-
*Total acids*	16.7 ± 2.4	1.16 ± 0.17	0.11 ± 0.00
Acetic acid	3.39 ± 0.03	1.16 ± 0.17	0.09 ± 0.00
Malic acid	2.13 ± 0.77	-	0.02 ± 0.00
Lactic acid	9.46 ± 1.44	-	-
Formic acid	1.72 ± 0.02	-	-

### The effect of Cellic® CTec3 (cellulolytic enzymes), AMG® 300 L BrewQ (amylolytic enzymes), and mixing of OFMSW with beechwood cellulose on sugar yield after EH

A full factorial experimental design was prepared to evaluate the effects of each variable as shown in Table 3. All trials were conducted in triplicates, except the central point (trial no.9), which was performed with five replicates. The experiment was performed on beechwood pulp batch 1. The results of the two-way ANOVA show that Ctec3 dosage has a significant effect on saccharification. Similarly, the interaction between Ctec3 dosage and feedstock composition in the form of OFMSW/cellulose ratio is also statistically significant (*F* = 14.1, *p* = 0.001), suggesting that the effect of Cellic® CTec3 is influenced by the presence of varying cellulose in the feedstock. In contrast, the factors of AMG dosage and OFMSW/cellulose ratio, as well as their interactions, do not appear to impact the sugar yield significantly. Additionally, based on central points, no curvature was detected. All statistics are provided in Table S1, in Supplementary Information.

**Table 3:** Sugar release from OFMSW/beechwood cellulose biomasses under different dosages of Ctec3 and AMG, based on full factorial design

Trial	Factors			Response
	FPU of CTec3/g of biomass DM	AGU of AMG/g of biomass DM	OFMSW inclusion (%)	Sugar yield (g/100 g biomass DM)
1	10	5	20	44.8 ± 1.9
2	10	0.5	20	40.7 ± 1.9
3	1	5	20	25.9 ± 6.0
4	1	0.5	20	29.6 ± 4.1
5	10	5	80	36.8 ± 0.9
6	10	0.5	80	37.0 ± 2.1
7	1	5	80	34.9 ± 7.7
8	1	0.5	80	33.1 ± 2.8
9	5,5	2.75	50	38.3 ± 1.7

### The effect of CTec3 and OFMSW inclusion on saccharification of beechwood cellulose from batch 1 and batch 2

Based on the outcomes presented in the “The effect of Cellic® CTec3 (cellulolytic enzymes), AMG® 300 L BrewQ (amylolytic enzymes), and mixing of OFMSW with beechwood cellulose on sugar yield after EH” section, the decision was reached to conduct a more in-depth study into the impacts of Ctec3 dosage and feedstock composition. This decision was influenced by the fact that altering the dosage of AMG did not yield any statistically significant changes in sugar yield. This may be due to the relatively low starch content in the feedstock mixtures, which ranged from 12 to 3 g of starch per 100 g of biomass DM, making the low dosage of AMG not a limiting factor during saccharification. Consequently, it was determined that this factor should be maintained at a consistent level of 0.5 AGU AMG per g of feedstock DM. With this intention, the experimental setup was devised to assess the effects of Ctec3 dosage and feedstock composition on saccharification. Exact numerical results are in Table S2 and Table S4, in Supplementary Information.

MLR analysis was performed to investigate how Ctec3 dosage and OFMSW/cellulose ratio influence sugar yield as a response variable. The model explains a substantial portion of the variability in concentration, with an adjusted *R*^2^ of 0.88. Statistics from the model are presented in Table S3, in Supplementary Material. The squared term of the OFMSW variable has a coefficient of − 41.0. This indicates a curved relationship between the OFMSW/cellulose ratio and sugar yield. Initially, as OFMSW/cellulose ratio increases, sugar yield rises, but at a certain point, further increases in OFMSW content lead to a decrease in sugar concentration. Specifically, as the OFMSW content in the beechwood cellulose mixture increases, the sugar yield also increases, reaching a peak at around 45 w/w% of OFMSW in the mixture. However, adding more OFMSW beyond this point leads to a decline in sugar yield.

**Fig. 1:**
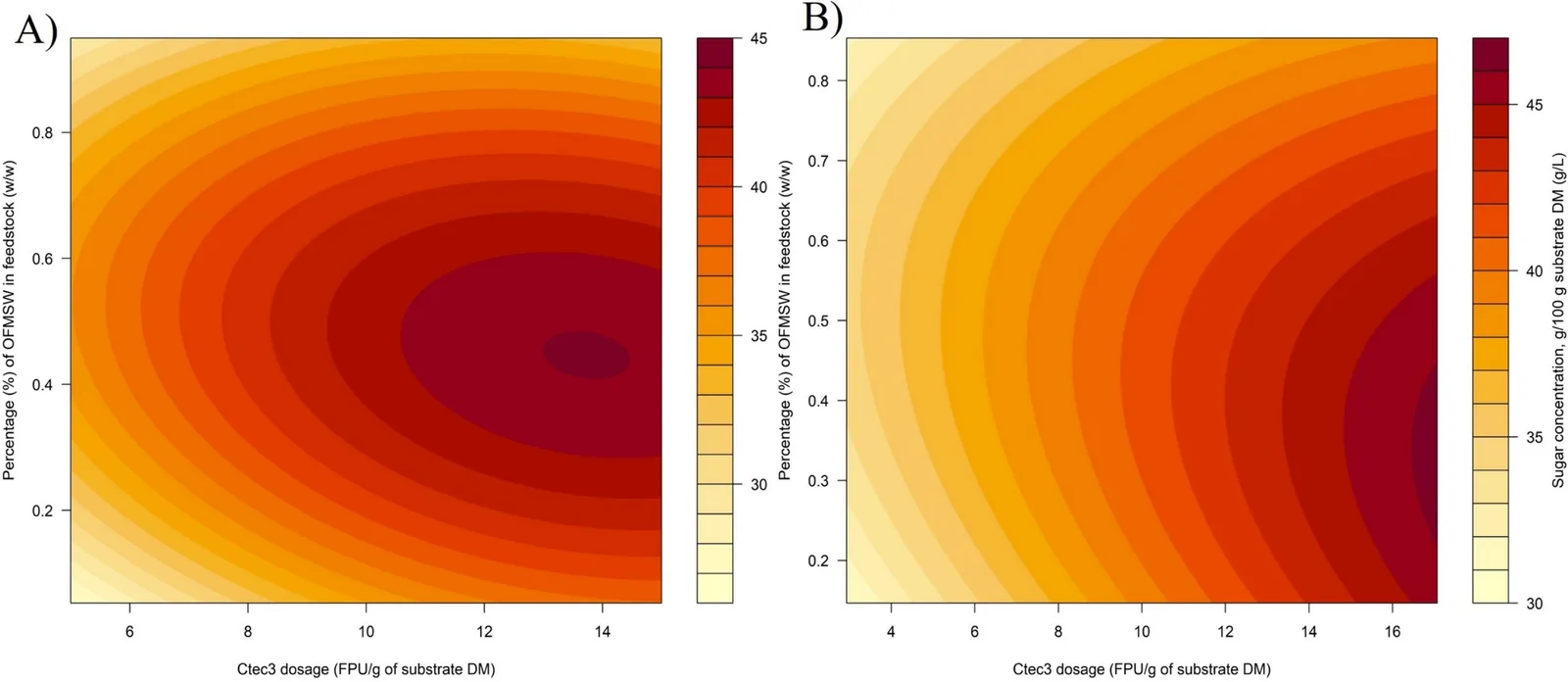
**A** The contour plot based on the MLR model of saccharification of OFMSW and beechwood cellulose batch 1 mixtures. **B** The contour plot based on the MLR model of saccharification of OFMSW and beechwood cellulose batch 2 mixtures. The key plots are represented by hydrolyzed sugars g/100 g of substate DM

The interaction between Ctec3 dosage and OFMSW inclusion is captured by the coefficient value of − 0.813. This negative coefficient suggests that the impact of Ctec3 dosage on sugar yield is reduced as the level of OFMSW inclusion increases. The contour plot in Fig. 1A illustrates how Ctec3 dosage and OFMSW/cellulose ratio impact the saccharification process, showing a quadratic interaction between OFMSW/cellulose ratio and sugar yield. The optimum for saccharification is predicted to be at ~ 45 w/w% of OFMSW inclusion for batch 1.

The application of batch 2 aimed to identify the robustness and predictability of the enzymatic hydrolysis process towards varying compositions of beechwood cellulose. Considering the results from batch 1, it was determined that the optimal addition of OFMSW into cellulose will be at 25–75 w/w% w/w inclusion. Based on queried knowledge, an experiment with central composite design (CCD) was conducted to identify saccharification of beechwood cellulose from batch 2. All trials were conducted in triplicates, except the central point, which was performed in five replicates. The CCD results were used to make the MLR model, and the response surface model (RSM) was visualized in the form of a contour plot in Fig. 1B. Similarly to results for batch 1, a quadratic effect is observed with the prediction of saccharification optimum at ~ 35 w/w% of OFMSW inclusion for batch 2 of beechwood cellulose. More precisely, as the OFMSW content in the beechwood cellulose mixture increases, the sugar yield also increases, reaching a peak at around 35 w/w% of OFMSW in the substrate mixture. However, adding more OFMSW beyond this level results in a decrease in sugar yield. Statistics from the model are presented in Table S5, in Supplementary Material.

### The comparison of saccharification of pure organosolv beechwood and mixtures of OFMSW with batch 1 and batch 2

**Fig. 2:**
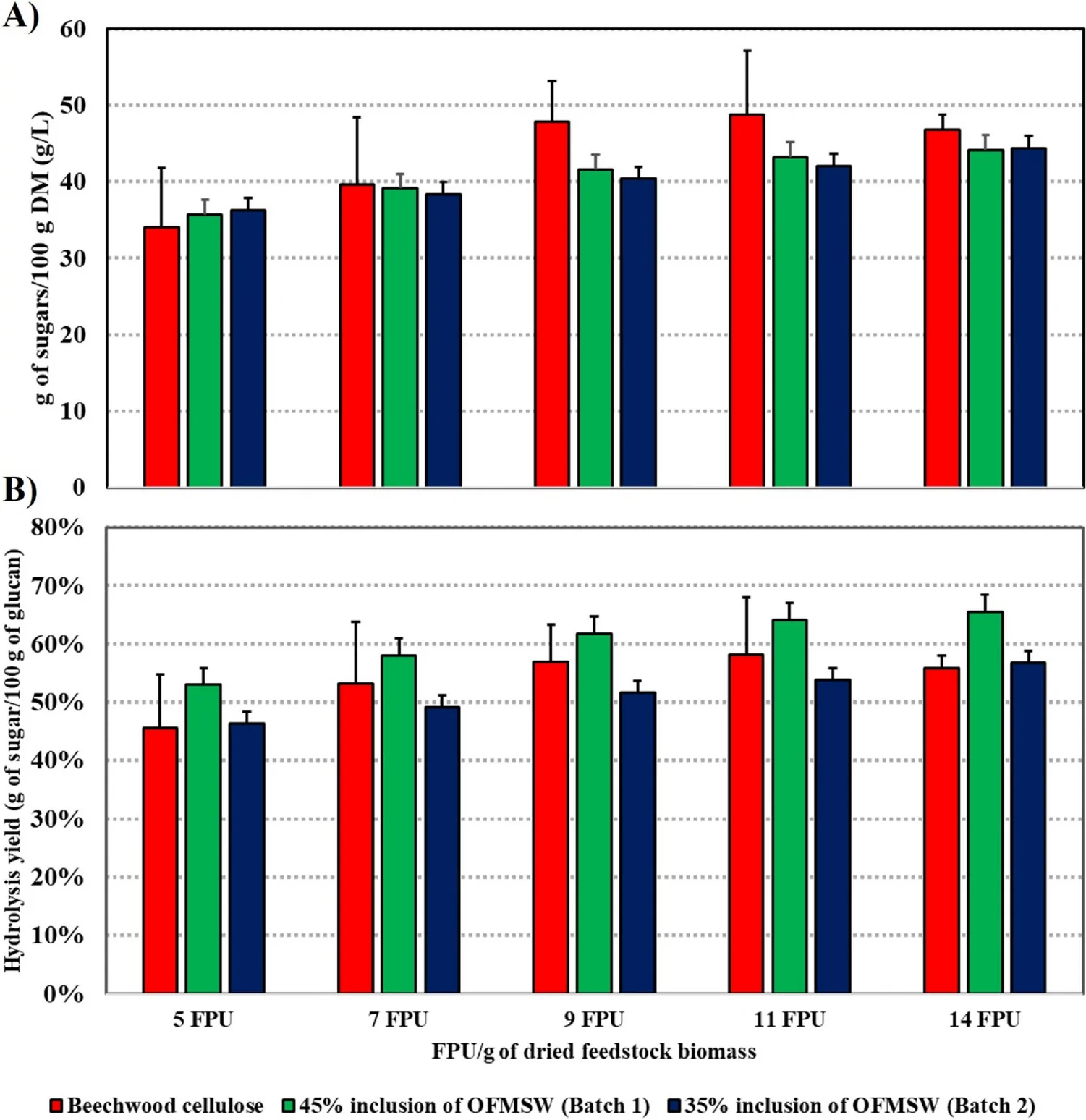
The comparison of sugar yields from pure beechwood pulp and mixtures of OFMSW with batch 1 and batch 2. Results are presented for **A** g of sugar/100 g of feedstock DM and for **B** g of sugar/100 g of glucan in feedstock DM

The sugar yield achieved through the hydrolysis of pure beechwood cellulose was compared to those predicted sugar yields using MLR models for the hydrolysis of mixtures containing both OFMSW and beechwood cellulose. These predictions were generated with OFMSW inclusions set at their estimated optimal levels, specifically 45% and 35%, for both batch 1 and batch 2 of beechwood cellulose. The findings illustrated in Fig. 2A indicate that the dosage of the Ctec3 enzyme influences the sugar yield obtained in the EH process. Furthermore, as the enzyme dosage increases, the sugar yield begins to plateau for all types of feedstock. Interestingly, when OFMSW is introduced into the mixture, it leads to proportional sugar concentrations when compared to pure beechwood cellulose. These results highlight the possibility of achieving similar sugar yields by incorporating OFMSW into beechwood pulp cellulose. Additionally, when comparing the sugars obtained from hydrolysis to the total sugar content in the feedstocks Fig. 2B, it becomes evident that the combination of OFMSW and beechwood cellulose exhibits superior saccharification efficiency, achieving a conversion rate of over 65%, as opposed to beechwood cellulose alone, which achieves a conversion rate of just over 58%.

### Ethanol fermentation

**Fig. 3:**
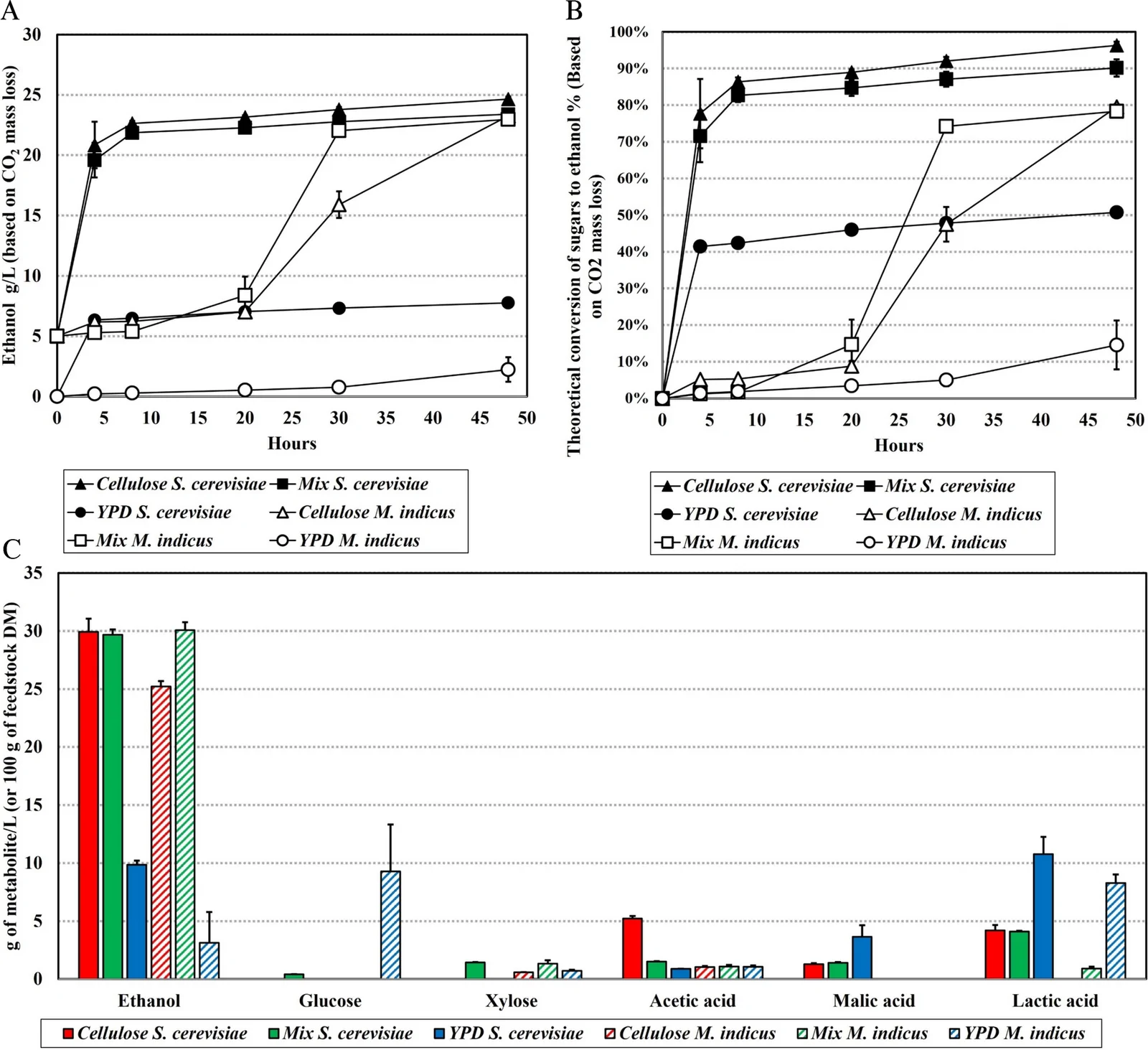
**A** Ethanol production achieved through the cultivation of *S. cerevisiae* and *M. indicus* using beechwood cellulose hydrolysates, OFMSW/beechwood cellulose hydrolysate, and YPD media. **B** Conversion of sugar to ethanol during the cultivation of *S. cerevisiae* and *M. indicus* on beechwood cellulose hydrolysates, OFMSW/beechwood cellulose hydrolysate, and YPD media. **C** HPLC analysis of ethanol, residual sugars, and organic acids in fermented cellulose hydrolysate, OFMSW/cellulose hydrolysate, and YPD media via *S. cerevisiae* and *M. indicus*. In the figure, red bars indicate the fermentation of cellulose hydrolysate, green bars indicate the fermentation of OFMSW/cellulose mixture hydrolysate, and blue bars indicate the fermentation of YPD media. Full bars represent media fermented with *S. cerevisiae*, and patterned bars represent media fermented with *M. indicus*

The findings derived from the CO_2_ mass loss during ethanol production, as depicted in Fig. 3A, demonstrate that beechwood cellulose hydrolysates supplemented with nitrogen exhibited ethanol production levels in the range of 23 to 25 g/L of hydrolysate, closely matching those achieved with the enzymatically hydrolyzed mixture of beechwood cellulose and OFMSW (with 40 w/w% inclusion) after a 48-h fermentation period for both fermentative microorganisms. In contrast, YPD media showed poor results for ethanol production, with only 7.8 g of ethanol/L of medium for *S. cerevisiae* and an insignificant 2.2 g of ethanol/L of medium for *M. indicus*. Comparing the ethanol fermentation performance of *S. cerevisiae* and *M. indicus*, it is evident that *S. cerevisiae* metabolizes glucose in hydrolysates more rapidly than *M. indicus*, with a plateau achieved between 4 and 8 h into the fermentation process. For instance, *M. indicus* reaches a plateau at approximately 30 h into the fermentation when utilizing OFMW/cellulose hydrolysates, but when cellulose hydrolysates are fermented, this plateau is reached later, spanning from 30 to 48 h into the fermentation. Furthermore, with regard to the conversion of sugar to ethanol in beechwood cellulose and mixture hydrolysates, as illustrated in Fig. 3B, it is observed that *S. cerevisiae* utilizes the majority of the available glucose for ethanol production, given its capability to metabolize glucose exclusively. In contrast, *M. indicus* converts slightly less than 80% of the available sugars into ethanol, as it can ferment glucose and xylose. In this case, the difference in sugar utilization efficiency makes *M. indicus* less effective in sugar-to-ethanol fermentation, whether in hydrolysates or YPD media. Furthermore, results show that *M. indicus* exhibits a significantly longer adaptive lag-phase than *S. cerevisiae.*

HPLC analysis of fermentation broths, shown in Fig. 3C, confirmed the achieved production of ethanol based on the assessment of CO_2_ mass loss. Interestingly, the results of HPLC analysis in Fig. 3C showed higher ethanol concentrations than initially anticipated based on CO_2_ mass reduction in Fig. 3A, showing a discrepancy of up to 7 g of ethanol/L for the fermentation of OFMSW/beechwood cellulose hydrolysate with both *M. indicus* and *S. cerevisiae*, reaching up to 30 g of ethanol/L. Similar outcomes were observed in our previous study with OFMSW enzymatic hydrolysates (Rudnyckyj et al. 2023). This difference could be explained by the increased presence of lactic acid in the broths, indicating the potential presence of heterofermentative lactic acid bacteria during fermentation. These bacteria have the capacity to metabolize glucose into lactic acid/acetic acid and ethanol without generating CO_2_ as a byproduct. Moreover, lactic acid bacteria are known to have a symbiotic relationship with *S. cerevisiae*, making it more likely to be the cause of increased ethanol production. Notably, the sole samples that exhibit minimal deviation in ethanol concentration between CO_2_ mass loss results and HPLC analysis are those involving cellulose hydrolysate fermentation with *M. indicus*. Intriguingly, no lactic acid was detected in these specific samples, thereby confirming the idea that the surplus ethanol is likely attributed to the presence of heterofermentative lactic acid bacteria.

The results suggest that OFMSW can serve as a promising substitute for artificially added nitrogen in ethanol fermentation, achieving comparable ethanol yields. Additionally, the similar ethanol production by both microorganisms confirms that sugar yields following the enzymatic hydrolysis of beechwood cellulose and the combination of OFMSW and beechwood cellulose are comparable.

### Cell biomass production

**Fig. 4:**
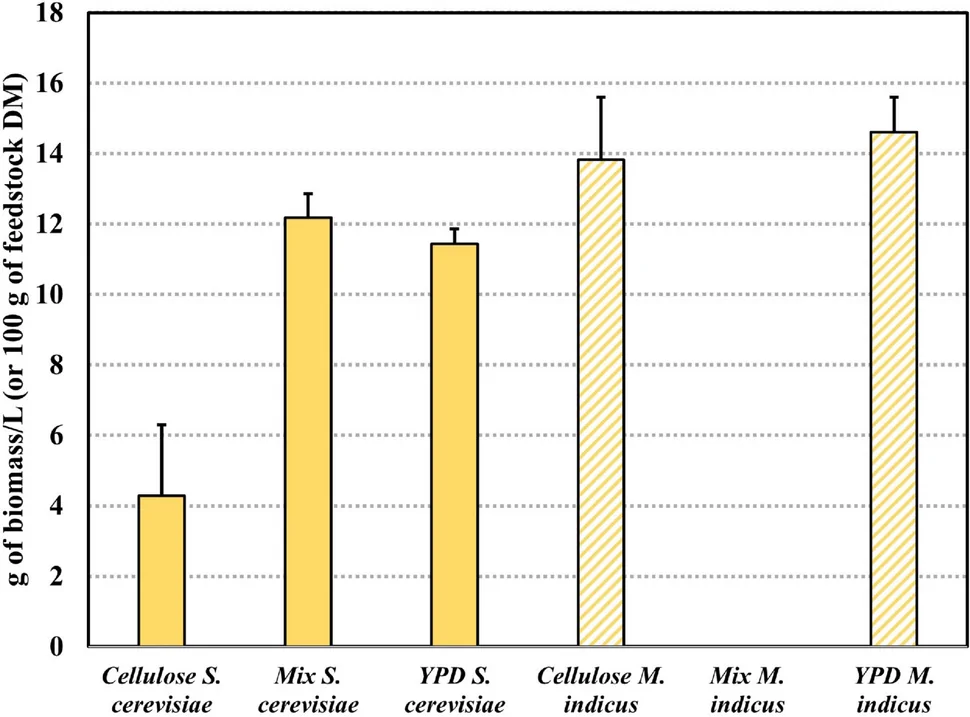
Cell biomass production via the cultivation of *S. cerevisiae* and *M. indicus* using beechwood cellulose hydrolysates, OFMSW/beechwood cellulose hydrolysate, and YPD media. Full bars represent media fermented with *S. cerevisiae*, and patterned bars represent media fermented with *M. indicus*

The findings presented in Fig. 4 show that hydrolysate produced from the mixture of 40% OFMSW and 60% beechwood pulp (w/w) yields a high amount of 12.2 g of cell biomass or dry cell weight/100 g feedstock DM or 0.305 g of biomass/g of carbon source, followed by YPD media and then beechwood pulp hydrolysate for fermentation with *S. cerevisiae*. On the other hand, *M. indicus* did not yield cell biomass when utilized in the OFMSW/cellulose mixture hydrolysate but displayed more efficient performance with cellulose hydrolysate and YPD media compared to *S. cerevisiae*, reaching 14.6 and 13.8 g/100 g of feedstock DM, respectively. The HPLC analysis of post-cultivation broths from the cultivation of *M. indicus* on OFMSW/cellulose mixture hydrolysate revealed the same sugar content as freshly hydrolyzed OFMSW/cellulose mixture, reaching 45 g of sugars/100 g feedstock DM. Based on this finding, it was suggested that the growth of *M. indicus* was inhibited in OFMSW/cellulose mixture hydrolysate during aerobic cultivation.

## Discussion

The compositional analysis of OFMSW has revealed a significant abundance of organic acids. This characteristic offers the advantage of imparting a buffering effect when combining OFMSW with beechwood cellulose. The composition of two beechwood cellulose batches shows that batch 1 has 75.9 ± 0.5 g glucan/100 g DM, and batch 2 has 97.5 ± 2.5 g glucan/100 g DM. These specific batches were deliberately selected to test the advantages derived from the incorporation of OFMSW into cellulose materials. Batch 1 represents the lower limit of cellulose content following organosolv extraction, while batch 2 stands at the upper limit of cellulose content attainable through the same extraction process. This strategic choice enables a comprehensive evaluation of the effects of treating these cellulose batches, shedding light on the robustness and versatility of the proposed approach.

The initial screening of variables, including factors such as Ctec3 dosage, AMG dosage, and OFMSW inclusion, revealed that Ctec3 dosage has the most significant positive impact on enzymatic saccharification. In contrast, variations in AMG dosage showed no significant effect on the saccharification of the OFMSW and beechwood cellulose blend. This can be explained by the fact that there is a high content of cellulose and a relatively low content of starch in the feedstock mixture, ranging approximately from 74 to 43 g of cellulose and hemicellulose/100 g dried biomass and from 12 to 3 g of starch/100 g dried biomass. Based on these conclusions, the following experiments had a constant AMG dosage of 0.5 AGU/g of feedstock DM and varying Ctec3 dosage and OFMSW inclusion.

An investigation involving CCD and MLR models determined that optimal sugar yields were achieved by including 45 w/w% OFMSW in the cellulose of batch 1 and 35 w/w% OFMSW in the cellulose of batch 2. These percentages were identified as optimal for the hydrolysis process. To provide a meaningful comparison, the sugar yield obtained from the hydrolysis of pure beechwood cellulose, which necessitated the use of a 50 mM acetate buffer, was contrasted with the predicted sugar yields when utilizing 45 w/w% OFMSW inclusion for batch 1 and 35 w/w% OFMSW inclusion for batch 2 in Fig. 2. Remarkably, the results of this comparison revealed that the sugar yields achieved with the mixtures were comparable with those attained with pure beechwood cellulose. Based on these findings, it can be reasonably suggested that incorporating OFMSW into cellulosic material is a viable alternative to traditional buffering agents, as evidenced by its effective substitution for the 50 mM acetate buffer in this study. This demonstrates the potential for eco-friendly and cost-effective approaches to hydrolysis processes, with promising implications for sustainable bioprocessing.

Ethanol fermentation using hydrolysates exclusively derived from beechwood cellulose and the combination of 40% OFMSW and 60% beechwood cellulose on a dry mass basis exhibited exceptional sugar-to-ethanol conversion efficiency for both *S. cerevisiae* and *M. indicus*, resulting in ethanol concentrations ranging from 25 to 30 g of ethanol/L of hydrolysate. In comparison, Katsimpouras et al. (2017) demonstrated a high ethanol concentration of 75.9 g/L of hydrolysate through *S. cerevisiae* fermentation on enzymatically hydrolyzed delignified beechwood cellulose with high solids loading. Moreover, Kalogiannis et al. (2018) achieved 80 g of ethanol/L of organosolv beechwood cellulose hydrolysate, and de Vrije et al. (2024) obtained approximately 100 g of ethanol/L of organosolv beechwood cellulose hydrolysate with *S. cerevisiae* fermentation. However, these high ethanol concentrations were achieved due to the elevated glucose concentration in hydrolysate media. The overall sugar-to-ethanol conversion observed in the presented studies was comparable to that of this study, reaching over 80% of the maximum theoretical ethanol yield.

A comparison in the performance of *S. cerevisiae* and *M. indicus* highlights the fact that *S. cerevisiae* can rapidly convert sugars, while *M. indicus* exhibits a prolonged adaptive lag phase when dealing with hydrolysates. Similarly, Lennartsson et al. (2012) reported a prolonged lag phase during ethanol fermentation with *M. indicus* on orange peel hydrolysate. Moreover, the equivalent ethanol yields in the fermented hydrolysates confirm that the enzymatic hydrolysis of both beechwood cellulose and the mixture of OFMSW and beechwood cellulose achieved similar sugar yields. Surprisingly, *M. indicus* and *S. cerevisiae* had poor ethanol production in YPD media, but it can also be attributed to the fact that this synthetic media is used for cell growth, and the C/N ratio is considered too low to be optimal for ethanol production (Manikandan and Viruthagiri 2010).

Aerobic cultivation of *S. cerevisiae* demonstrated promising outcomes, with the highest biomass production achieved in the case of OFMSW/cellulose hydrolysate, followed by YPD media and cellulose hydrolysate supplemented with a nitrogen source. Notably, the cultivation of *S. cerevisiae* on OFMSW/cellulose hydrolysate resulted in a higher biomass yield compared to synthetic YPD media, which is commonly used for yeast growth. Additionally, OFMSW demonstrated itself to be an excellent substitute for synthetic nitrogen sources for *S. cerevisiae*, resulting in nearly threefold biomass production compared to cellulose hydrolysate supplemented with a synthetic nitrogen source. It could be suggested that OFMSW is not only a source of nitrogen but also essential and stimulating microbial growth factors such as minerals, vitamins, and co-factors.

In contrast, the aerobic cultivation of *M. indicus* did not result in biomass formation when using OFMSW/cellulose hydrolysate. Subsequent HPLC analysis revealed that the fermentation broths had a sugar content equivalent to a freshly hydrolyzed mixture of OFMSW/cellulose. This led to the conclusion that *M. indicus* experienced inhibited growth in OFMSW/cellulose hydrolysate under aerobic conditions. When cultivated on YPD media and cellulose hydrolysate with nitrogen supplementation, *M. indicus* achieved biomass yields of 14.6 and 13.8 g/100 g of feedstock DM, respectively.

Non-sterile conditions were intentionally selected to assess OFMSW's contamination control attributes, given its richness in antimicrobial compounds like organic acids, alcohols, and phenolics. Evidently, these conditions were advantageous for *S. cerevisiae* and *M. indicus*, with the exception of the inhibited growth of *M. indicus* under aerobic conditions. In addition, Dornau et al. (2020) reported that unhydrolyzed solids in OFMSW hydrolysates are rich in metals, which may cause additional growth inhibition. This highlights the need to prioritize the selection of microorganisms for OFMSW/cellulose mixtures based on their robustness to fully benefit from the antimicrobial properties of OFMSW.

In conclusion, the results suggest that OFMSW inclusion can effectively replace synthetic buffers in enzymatic conversion processes, achieving similar sugar yields. The optimal OFMSW addition may vary depending on specific conditions, but the current study found the range of 35–45 w/w% OFMSW in beechwood cellulose to be optimal. The fermentation of beechwood cellulose and OFMSW/cellulose mixtures resulted in efficient sugar-to-ethanol conversion for both *S. cerevisiae* and *M. indicus*, converting the majority carbon source into ethanol. Moreover, *S. cerevisiae* demonstrated excellent biomass production when aerobically cultivated in OFMSW/cellulose hydrolysate, surpassing YPD media. OFMSW also served as a substitute for synthetic nitrogen sources, boosting *S. cerevisiae* biomass production. Conversely, aerobic cultivation of *M. indicus* in OFMSW/cellulose hydrolysate completely inhibited biomass formation, potentially due to the high content of growth inhibitors in OFMSW and a prolonged lag phase of *M. indicus* in hydrolysates.

Subsequently, future research should emphasize the exploration of resilient microorganisms able to thrive in mixed biomass hydrolysates, achieving efficient utilization. Moreover, there is a need to extend investigations into the viability of incorporating various lignocellulosic materials alongside OFMSW as substrates. This will broaden the horizons and applicability of this eco-friendly approach to bioconversion processes. These research directions hold significant potential for advancing the sustainable evolution of bioprocessing technologies and facilitating the transition toward greener, more cost-effective biorefineries.

## Supplementary Information

Below is the link to the electronic supplementary material.

Supplementary file1 (PDF 133 KB)

## Data Availability

Data will be available upon request from the corresponding author.
