# Supplementary material for: Organic waste and beechwood cellulose blend saccharification and validation of hydrolysates by fermentation
Source: Appl Microbiol Biotechnol. 2024 Nov 14;108(1):517. doi: 10.1007/s00253-024-13349-2 (PMC11564323; doi:10.1007/s00253-024-13349-2)
Supplement: Supplementary file 1 — Supplementary file1 (PDF 133 KB) [file 253_2024_13349_MOESM1_ESM.pdf]

Organic waste & beechwood cellulose blend saccharification and validation of hydrolysates by fermentation

Journal name: Applied Microbiology and Biotechnology

Stanislav Rudnyckyj*[a], Sergey Kucheryavskiy[b], Tanmay Chaturvedi[a] and Mette Hedegaard Thomsen[a]
[a]Aalborg University, Department of Energy, Niels Bohrs Vej 8, 6700 Esbjerg, Denmark
[b]Aalborg University, Department of Chemistry and Bioscience, Niels Bohrs Vej 8, 6700 Esbjerg, Denmark
*stru@energy.aau.dk

Table S1: The two-way ANOVA analysis of the full factorial experiment with two levels of CTec3 dosage, AMG dosage, and OFMSW/cellulose ratio.

Factor	F-value	P-value
Ctec3 dosage	30.5	<0.001 *
AMG dosage	0.10	0.756
OFMSW inclusion	0.02	0.900
**Interaction between factors**		
Ctec3:AMG	0.77	0.389
Ctec3: OFMSW inclusion	14.1	0.001 *
AMG: OFMSW inclusion	0.03	0.855

"*" represents statistical significance

Table S2: The CCD with varying Ctec3 dosage and OFMSW inclusion and resulting sugar yields for beechwood cellulose Batch 1.

Trial	Factors		Response
	FPU of CTec3/g of biomass DM	OFMSW inclusion (%)	Sugar yield (g/100 g biomass DM)
1	15	35	43.1±1.0
2	5	35	35.8±2.8
3	15	5	37.9±0.7
4	5	5	25.3±0.2
5	15	65	41.6±3.0
6	5	65	35.1±1.0
7	15	95	33.8±2.4
8	5	95	29.1±0.3
9	10	20	40.7±1.9
10	10	80	37.0±2.1

Table S3: The statistics from the MLR model for CCD experiments for beechwood cellulose from Batch 1. The P-value and F-value of the model are 4.49×10-11 * and 41.8, with an adjusted R2 of 0.88.

Factor	Coefficients	Standard Error	t-value	P-value
(Intercept)	10.1	3.29	3.07	<0.001 *
Ctec3 dosage	3.39	0.734	4.62	<0.001 *
OFMSW inclusion	47.8	5.20	9.19	<0.001 *
**Interaction between factors**				
Ctec3: OFMSW inclusion	-0.813	0.293	-3.40	0.002 *
**Quadratic interaction**				
Ctec3	-0.110	0.036	-3.06	0.005 *
OFMSW inclusion	-41.0	4.47	-9.17	<0.001 *

"*" represents statistical significance

Table S4: The CCD with varying Ctec3 dosage and OFMSW inclusion and resulting sugar yields for beechwood cellulose Batch 2.

**Trial**	**Factors**		**Response**
	FPU of CTec3/g of biomass DM	OFMSW inclusion (%)	Sugar yield (g/100 g biomass DM)
1	15	25	36.5±2.5
2	5	25	45.6±1.0
3	15	75	35.2±0.4
4	5	75	40.7±0.2
5	17,07	50	45.5±0.8
6	2,93	50	33.8±1.0
7	10	85	38.3±1.1
8	10	15	36.8±0.4
9	10	50	41.3±0.5

Table S5: The statistics from the MLR model for CCD experiments for beechwood cellulose from Batch 2. The P-value and F-value of the model are 7.09×10^{-10} * and 33.9, with an adjusted R^2 of 0.85.

**Factor**	**Coefficients**	**Standard Error**	**t-value**	**P-value**
(Intercept)	22.9	3.23	7.08	<0.001 *
Ctec3 dosage	1.60	0.41	3.90	<0.001 *
OFMSW inclusion	29.8	8.23	3.62	0.001 *
**Interaction between factors**				
Ctec3: OFMSW inclusion	-0.71	0.37	-1.94	0.064
**Quadratic interaction**				
Ctec3	-0.02	0.02	-1.30	0.208
OFMSW inclusion	-26.5	7.25	-3.65	0.001 *

"*" represents statistical significance
